# Metabolomics Identifies Novel Blood Biomarkers of Pulmonary Function and COPD in the General Population

**DOI:** 10.3390/metabo9040061

**Published:** 2019-04-01

**Authors:** Bing Yu, Claudia Flexeder, Robert W. McGarrah, Annah Wyss, Alanna C. Morrison, Kari E. North, Eric Boerwinkle, Gabi Kastenmüller, Christian Gieger, Karsten Suhre, Stefan Karrasch, Annette Peters, Gregory R. Wagner, Gregory A. Michelotti, Robert P. Mohney, Holger Schulz, Stephanie J. London

**Affiliations:** 1 Department of Epidemiology, Human Genetics, and Environmental Sciences, School of Public Health, The University of Texas Health Science Center at Houston, Houston, TX 77030, USA; bing.yu@uth.tmc.edu (B.Y.); Alanna.C.Morrison@uth.tmc.edu (A.C.M.); Eric.Boerwinkle@uth.tmc.edu (E.B.); 2 Institute of Epidemiology, Helmholtz Zentrum München—German Research Center for Environmental Health, 85764 Neuherberg, Germany; claudia.flexeder@helmholtz-muenchen.de (C.F.); christian.gieger@helmholtz-muenchen.de (C.G.); stefan.karrasch@helmholtz-muenchen.de (S.K.); peters@helmholtz-muenchen.de (A.P.); 3 Division of Cardiology, Department of Medicine, Duke University School of Medicine, Durham, NC 27713, USA; robert.mcgarrah@duke.edu; 4 Epidemiology Branch, National Institute of Environmental Health Sciences, National Institutes of Health, Department of Health and Human Services, Research Triangle Park, NC 27709, USA; annah.wyss@nih.gov; 5 Department of Epidemiology, University of North Carolina, Chapel Hill, NC 27599, USA; kari_north@unc.edu; 6 Human Genome Sequencing Center, Baylor College of Medicine, Houston, TX 77030, USA; 7 Institute of Bioinformatics and Systems Biology, Helmholtz Zentrum München—German Research Center for Environmental Health, 85764 Neuherberg, Germany; g.kastenmueller@helmholtz-muenchen.de; 8 Research Unit of Molecular Epidemiology, Helmholtz Zentrum München—German Research Center for Environmental Health, 85764 Neuherberg, Germany; 9 Weill Cornell Medicine-Qatar, Department of Physiology and Biophysics, Education City, Doha, Qatar; kas2049@qatar-med.cornell.edu; 10 Comprehensive Pneumology Center Munich (CPC-M), Member of the German Center for Lung Research (DZL), 81377 Munich, Germany; 11 Institute and Outpatient Clinic for Occupational, Social and Environmental Medicine, Ludwig-Maximilians-Universität, 80336 Munich, Germany; 12 Discovery and Translational Sciences, Metabolon, Inc., Durham, NC 27713, USA; gwagner@metabolon.com (G.R.W.); GMichelotti@metabolon.com (G.A.M.); RMohney@metabolon.com (R.P.M.)

**Keywords:** metabolome, metabolomics, chronic obstructive pulmonary disease, respiratory function tests, forced expiratory volume

## Abstract

Determination of metabolomic signatures of pulmonary function and chronic obstructive pulmonary disease (COPD) in the general population could aid in identification and understanding of early disease processes. Metabolome measurements were performed on serum from 4742 individuals (2354 African-Americans and 1529 European-Americans from the Atherosclerosis Risk in Communities study and 859 Europeans from the Cooperative Health Research in the Region of Augsburg study). We examined 368 metabolites in relation to cross-sectional measures of forced expiratory volume in 1 s (FEV1), forced vital capacity (FVC), their ratio (FEV1/FVC) and COPD using multivariable regression followed by meta-analysis. At a false discovery rate of 0.05, 95 metabolites were associated with FEV1 and 100 with FVC (73 overlapping), including inverse associations with branched-chain amino acids and positive associations with glutamine. Ten metabolites were associated with FEV1/FVC and seventeen with COPD (393 cases). Enriched pathways of amino acid metabolism were identified. Associations with FEV1 and FVC were not driven by individuals with COPD. We identified novel metabolic signatures of pulmonary function and COPD in African and European ancestry populations. These may allow development of biomarkers in the general population of early disease pathogenesis, before pulmonary function has decreased to levels diagnostic for COPD.

## 1. Introduction

Pulmonary function, as assessed by forced expiratory volume in 1 s (FEV1), forced vital capacity (FVC), and the ratio of FEV1 to FVC (FEV1/FVC), reflects the physiological state of the lung. These measures are used to diagnose and monitor chronic obstructive pulmonary disease (COPD). Reduced pulmonary function in the general population correlates with systemic inflammation biomarkers [1,2] and has been linked to development of other pathologies including diabetes [3], kidney [4] and cardiovascular diseases [5,6]. Pulmonary function is influenced by genetics [7,8] and environmental factors, most notably, tobacco smoking [9]. Lower pulmonary function is a risk factor for mortality in the general population, independently of smoking and other risk factors and even among individuals with normal spirometry [10,11,12,13]. However, the underlying mechanisms for these diverse impacts of reductions in pulmonary function remain unknown.

Metabolomics systematically identifies and quantifies small molecules in blood and other biologic tissues. It has increasingly been applied to the study of chronic diseases [14,15,16,17]. Various circulating metabolites have been reported as associated with COPD in several clinically selected studies, generally with a modest number of cases [18,19,20,21,22,23,24,25,26,27,28,29,30]. Studies of COPD identified in clinical settings identify more severe disease than population-based studies. Studies of more severe disease may tend to identify markers of disease progression and treatment. The clinical utility of identifying circulating biomarkers of very early stages of COPD has been highlighted [31]. Biomarkers of the earliest stages of the disease process can enable development of new therapies and improve understanding of mechanisms by which reduced pulmonary function contributes to risk of developing chronic diseases. Well-powered studies of circulating metabolites in relation to spirometric measures in general population samples, unselected for disease, can identify reproducible biomarkers of early stages of the disease process before pulmonary function has declined to levels diagnostic of airflow obstruction and COPD. Publications on circulating metabolites in relation to pulmonary function in population cohorts are rare. A study of UK twins identified metabolites, including several amino acids, cofactors and vitamins, related to both FEV1 and FVC, as well as some unique associations for each [32,33]. No study has examined the association of metabolomic profiles with pulmonary function as well as relationships with COPD in a multi-ethnic general population sample.

We conducted meta-analyses of metabolomic analytes in relation to pulmonary function (FEV1, FVC and FEV1/FVC) in 2354 individuals of African ancestry and 2388 individuals of European ancestry from the Atherosclerosis Risk in Communities (ARIC) study and Cooperative Health Research in the Region of Augsburg (KORA). We further examined molecular pathways of specific metabolites and associations of metabolites with COPD.

## 2. Results

### 2.1. Study Characteristics

Characteristics of study participants are presented in Table 1. Mean ages were very similar for the 2354 ARIC African-Americans (53.0 years, SD = 5.7), the 1,529 ARIC European-Americans (54.6, SD = 5.8) and the 859 KORA Europeans (53.8, SD = 5.7). Current smoking prevalence were also similar, ranging from 21 to 28%. A total of 393 individuals met our definition of COPD (358 from ARIC and 35 from KORA).

### 2.2. Metabolic Associations with Pulmonary Function Measures

**Figure 1:**
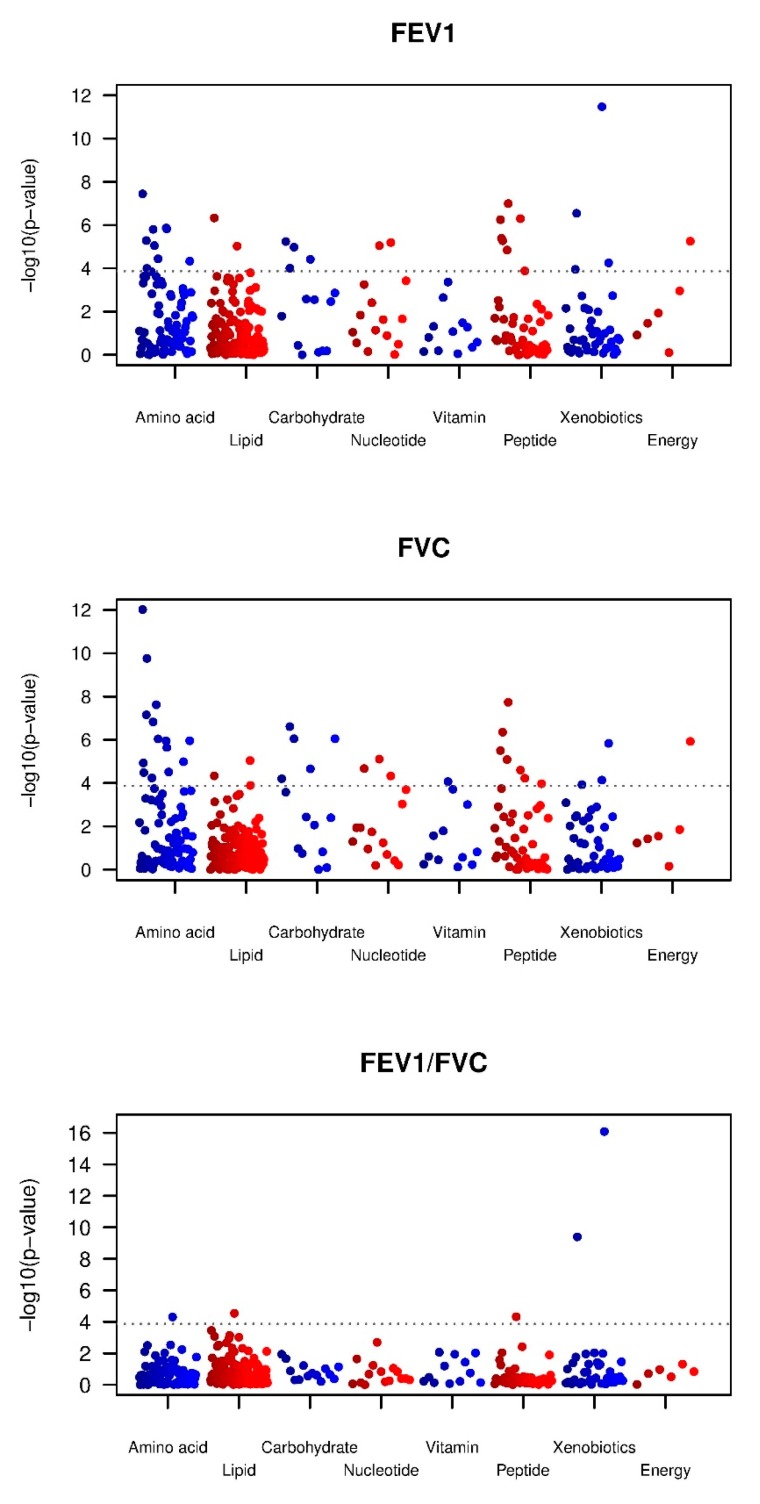
Manhattan plots for metabolome-wide association *p*-values for the three pulmonary function parameters in meta-analysis. The X axis indicates metabolite classes (super pathways) and Y axis indicates –log(10) *p*-values for each metabolites.

Manhattan plots for the three pulmonary function parameters are shown in Figure 1. At FDR < 0.05, 95 metabolites were associated with FEV1 (30 at Bonferroni correction) and 100 (37 at Bonferroni correction) with FVC. Given the large number of findings, in the main text tables, we show only the top 20 associations (based on meta-analysis *p* value) that meet Bonferroni correction where the metabolite was measured in all three datasets (Table 2 for FEV1 and Table 3 for FVC); all metabolites associated at FDR < 0.05 are in Table S1 for FEV1 and Table S2 for FVC. For metabolites associated at FDR < 0.05, the average effect size per SD difference in metabolite for FEV1 was a 25.8 mL difference (range 17.3 mL–50.7 mL) and for FVC, a 29.9 mL difference (range 19.7 mL–56.9 mL). There were 23 metabolites associated, at Bonferroni significance, with both FEV1 and FVC, all with matching directions of effect. These include eight amino acids and derivatives (glycine, asparagine, tryptophan betaine, 3-(4-hydroxyphenyl)lactate, 3-phenylpropionate (hydrocinnamate), isoleucine, 2-methylbutyrylcarnitine (C5) and glutamine), three carbohydrates (fructose, lactate and mannose), the lipid glycerol, the nucleotide N2,N2-dimethylguanosine, and several gamma-glutamyl amino acids. At FDR < 0.05, 73 metabolites associated with both FEV1 and FVC, all with matching directions (Table S3).

For FEV1/FVC we identified fewer associations (Table 4): 10 at FDR < 0.05, including 5 at Bonferroni significance. On average, per SD change of those 10 FDR significant metabolites is correlated with 0.48% difference (range 0.33% to 0.93%) in the FEV1/FVC, expressed as a percentage. Among the findings are two xenobiotics with inverse associations that reflect behaviors of individuals with low FEV1/FVC. These include the respiratory medication theophylline and its metabolite, 1,3-dimethylurate. Beside theophylline treatment in asthma and COPD patients, caffeine intake has to be considered as a common source for circulating theophylline. In sensitivity analyses of the association between metabolites and pulmonary function after deleting COPD cases, associations between FEV1/FVC and theophylline and its metabolites disappeared. These sensitivity analyses further showed that results for FEV1 and FVC were not driven by individuals with COPD: correlations between beta coefficients from fixed effect meta-analyses before and after exclusion of COPD were 0.96 for FEV1 and 0.99 for FVC.

### 2.3. Pathway Analyses

In pathway analyses, 83 out of 95 metabolites related to FEV1 and 84 of 100 related to FVC (FDR < 0.05) were matched to the pathway database and mapped to 49 and 42 pathways, respectively, for testing of enrichment. Among the four enriched pathways identified for either FEV1 or FVC, “aminoacyl-tRNA biosynthesis” and “phenylalanine metabolism” were implicated for both phenotypes (Table 5). We did not perform pathway analysis for FEV1/FVC given the small number of significant associations.

### 2.4. Metabolic Associations with COPD

For COPD, 17 metabolites were associated at FDR < 0.05, including five at Bonferroni significance (Table 6). On average, per SD difference of those 17 FDR significant metabolites, the odds of COPD changed by 22% (range 17% to 39%). Of the 17 metabolites associated with COPD at FDR < 0.05, only three (ornithine, homocitrulline, and 5-dodecenoate (12:1n7)) were not also identified at FDR < 0.05 for either FEV1, FVC or FEV1/FVC. For the 73 metabolites related to both FEV1 and FVC at FDR < 0.05 (Table S3); 23 were at least nominally associated with COPD (P < 0.05), including 8 at FDR < 0.05. Aside from theophylline and its metabolite, the other 6 metabolites were 3-(4-hydroxyphenyl)lactate (OR = 1.28, 95% CI: 1.14–1.44), glycerate, glycerol, 7-alpha-hydroxy-3-oxo-4-cholestenoate (7-Hoca), pseudouridine and serotonin (5HT).

### 2.5. Consistency of Associations

Directions of effect were extremely consistent across the three datasets for metabolites that were statistically significant in the meta-analysis. Across all associations at FDR < 0.05 for FEV1, FVC or FEV1/FVC, only a very small proportion of associations have directions of effect that do not match for all three datasets with the meta-analysis result (14 for FEV1, 10 for FVC and 0 for FEV1/FVC); the majority of the apparently discordant results are in the smallest dataset, KORA (Table S8). For COPD all 3 discordant results are again from KORA which contributed only 9% of COPD cases (Table S8). As expected, for all instances where the direction is flipped, the estimate is very imprecise with P-values ranging between 0.16 and 0.99 and a mean of 0.66 (Table S8). Significant results in the meta-analysis reflect that the fact that larger, more precisely estimated effects in a consistent direction in the other studies are not negated by a low precision estimate in the other direction. Results were also consistent between African and European ancestry populations. We did not identify any metabolites significantly related in the meta-analysis to any of the four traits (FDR < 0.05) where there was even a nominally significant association (uncorrected P < 0.05) in African-Americans where the effect estimate was in the opposite direction.

## 3. Discussion

By combining data from over 4700 individuals across two population-based studies, encompassing both African-American and European ancestry, we identified numerous metabolites related to quantitative pulmonary function measures. Individual metabolites related to FEV1 or FVC highlighted four enriched pathways that collectively reflect associations with amino acids and their gamma-glutamyl derivatives.

There have been various studies of circulating metabolomic profiles in relation to pulmonary function or COPD, predominantly in individuals selected in clinical setting for COPD phenotypes, including exacerbations [18,28,34,35,36]. Most included fewer than 100 individuals with COPD and several included less than 20. In clinically selected samples, it can be difficult to enroll a suitable control group truly representative of the population that gave rise to those cases. Given the large number of metabolites examined and the limited sample size of most studies, correction for multiple testing for individual metabolites was often not possible, making replication difficult. Circulating biomarkers of early COPD have been lacking and could be useful in development of interventions to limit disease progression. Examination of variations in circulating metabolites related to pulmonary function in the general population hold the potential to develop circulating markers of early stages of COPD pathogenesis that predate diagnostic reductions in pulmonary function [31]. However, studies of metabolomics profiles and quantitative pulmonary function traits across the general population are scarce. Our findings generally reflect associations across the normal range of these parameters, as they were largely similar when deleting individuals with COPD, despite the resulting smaller sample size and attenuation of the range of pulmonary function. A previous study by Menni et al. [32] with wide coverage (280 metabolites) in a population-based cohort of twins in the UK, identified at Bonferroni significance, 18 metabolites related to FEV1 and 10 related to FVC. From the combined list of 21 metabolites identified for either trait, we replicate 11 in our meta-analysis at FDR < 0.05 (3-phenylpropionate, asparagine, gamma-glutamylvaline, glycerate, glycine, indolepropionate, N-acetylglycine, pseudouridine, scyllo-inositol, succinylcarnitine, and threonate) and an additional three at nominal significance (serine, proline and glutamate). Four of their 21 metabolites were not included in our meta-analysis (butylcarnitine, C-glycosyltryptophan, CMPF-3-carboxy-4-methyl-5-propyl-2-furanpropanoate and pyroxidate). Three metabolites identified by Menni et al. did not replicate in our data (alpha-tocopherol, benzoate and bilirubin(ZZ)). However, gamma-tocopherol, which is tightly inversely correlated with alpha-tocopherol [37] and also strongly influenced by supplement use, was associated with FVC at FDR < 0.05 in the corresponding opposite direction from their alpha-tocopherol association. In addition, biliverdin, which, like bilirubin, is a product of heme breakdown, was related to both FEV1 and FVC at FDR < 0.05 in the same direction as their bilirubin association. Thus, our data provide very substantial replication of the population-based cohort findings of Menni et al. [32]. In addition to replication of previous findings, our meta-analysis identified many novel associations not reported by Menni et al. [32]. We identified at Bonferroni significance, 30 novel associations for FEV1 and 37 for FVC, including 22 that overlap (Table S3). At FDR < 0.05, we identified 111 novel metabolites related to either FEV1 or FVC (included in Tables S2 and S3). The effect sizes we observed for metabolites are not trivial in the context of the natural history of pulmonary function traits. Once maximum lung growth has been achieved, between 20–30 years of age, FEV1 and FVC inexorably decline with age. The mean effect size across significant associations, per SD difference in metabolite, of 25.8 mL, is greater than the estimated average decline in lung function among health nonsmoking adults of 18–20 mL per year [38]. It is also larger than the 10 year impact on FEV1 of exposure to secondhand tobacco smoke, estimated at 15 mL [39]. Modest differences can be meaningful because lower FEV1 and FVC at a given age increases the risk of declining below threshold levels diagnostic of COPD and lower lung function, even within the normal range is related to increased mortality, independently of other risk factors.

For FEV1/FVC, we identified far fewer associations than for either FEV1 or FVC. Identification of many more metabolites associated with FEV1 or FVC than FEV1/FVC suggests that our findings reflect coordinate effects on either preservation or deterioration in lung size as opposed to airflow obstruction. Metabolites significantly associated with FEV1 substantially overlapped with those for FVC with matching direction and similar magnitudes of effects. Accordingly, for the ratio of FEV1 to FVC, these associations generally canceled out. Although we identified many metabolites associated with FEV1 and FVC, speculation regarding mechanisms for most of these associations is difficult because the determinants of variation between individuals in the general population are not known for most metabolites. Experimental studies with manipulation of diet, physical activity, and other lifestyle factors in humans and animal models will be needed to gain a full understanding of normal inter-individual variation.

Among the interesting novel associations with FEV1 and/or FVC in our study is a cluster of metabolites previously associated with metabolic health. This cluster—containing the branched-chain amino acids (BCAA: isoleucine, leucine and valine), BCAA metabolic byproducts (2-methylbutyrylcarnitine, 3-hydroxyisobutyrate), aromatic amino acids (tyrosine, phenylalanine), glycine and glutamate/glutamine—was initially described as a metabolic signature in obese individuals that correlates with insulin resistance [40]. Subsequent studies demonstrated that this metabolic signature is prognostic for incident type 2 diabetes and predicts response to weight loss interventions [41,42,43,44]. Notably, the overrepresented pathways associated with FEV1 and FVC are enriched with these metabolites, as well. In general, higher BCAA/metabolic byproduct and aromatic amino acid concentrations, and lower glycine concentrations, reflect worse metabolic health. Thus, the inverse associations of BCAA/metabolic byproducts and aromatic amino acids, and positive association of glycine, with FEV1 and FVC, suggest that these metabolites might be reporting on the metabolic health status of individuals which, in turn, may impact pulmonary function. The glutamyl amino acids gamma-glutamylglutamine, gamma-glutamylglutamate, gamma-glutamylleucine and gamma-glutamylvaline were also associated with FEV1 and/or FVC, which likely reflects abundance of their cognate amino acids. Interestingly, a prior study of serum metabolomics in the ECLIPSE COPD cohort showed a decrease, rather than increase, in BCAA/metabolic byproducts in individuals with GOLD stage IV COPD compared with control individuals [36]. No association was seen with less severe COPD and similar findings have been seen in smaller clinically selected COPD studies [30,45]. In ECLIPSE, circulating glutamine, which largely reflects muscle turnover, was higher in COPD patients than controls [36], whereas in our general population study glutamine was positively associated with pulmonary function. Glutamine, the most abundant amino acid in the circulation, is a precursor for synthesis of glutathione, a major endogenous antioxidant, and plays important roles in response to injury and immune function [46,47]. Because patients with advanced COPD often exhibit reduced caloric intake, some degree of muscle wasting or frank cachexia, findings for BCAA and glutamine in these studies can be ascribed to the generalized state of protein deficiency and/or increased protein turnover [36].

A recent paper that examined urinary metabolites identified associations with histidine in relation to FEV1 and FEV1/FVC [48]. Although histidine was measured in our serum samples, we did not find associations with any of our phenotypes. Low lung function is associated with asthma and forms the basis for diagnosis of COPD. Thus, one may speculate, that biomarkers associated with low lung function may also be associated with lung disease. Three of the four major pathways enriched for both FEV1 and FVC in our analysis, “aminoacyl-tRNA biosynthesis” and “nitrogen metabolism” and “alanine-aspartate-glutamate metabolism”, were found to be associated with asthma phenotypes in 6–14 year old children [49]. Different carnitine compounds and amino acids, e.g., tyrosine, glutamine, glycine, asparagine, phenylalanine, leucine, and valine, as well as succinate have been associated with COPD in clinical studies [25,36] and were also detected in our analysis.

A recent study of 136 individuals from the COPDGene study also found differences in amino acids and to be related to exacerbations severity and frequency [50]. We are not able to examine exacerbations. However, the fact that we identified many of these associations in relation to quantitative traits of FEV1 and FVC, and these associations were not driven by individuals with COPD in our study, and that COPD in our study was skewed toward less severe disease than COPDGene, suggests that our findings are not impacted by failure to account for exacerbations.

Identifying subtle metabolic differences related to spirometry in the general population may shed light on why variation in pulmonary function, even within the normal range, is related to development of metabolic and cardiovascular disease outcomes [3,4,5,6]. Of interest, a recent study identified alterations in metabolic activity in cardiac tissue related to FEV1/FVC [51]. With respect to early metabolic changes that may presage COPD, in an experimental model of emphysema in the mouse, circulating isoleucine and valine were increased very early in the disease process [52] consistent with the inverse associations we observed for these branched chain amino acids and pulmonary function parameters in our general population study. Some recent studies have suggested the importance of sphingolipids and sphinomyelins in subphenotypes of COPD [18,29,45]. As noted, to be included in the meta-analysis, metabolites had to have measured in at least two of the three datasets. Because the three datasets were not measured at the same calendar time, the number of metabolites reported is greater in the newer datasets. No sphingolipids or sphingomyelins were reported for the KORA, thus our meta-analysis included only two sphingomyelins, both measured in ARIC: stearoyl sphingomyelin which was inversely associated with FVC in our data (FDR < 0.05) and palmitoyl sphingomyelin which was not associated with any phenotypes. In a previous study [29], sphingomyelins were related to emphysema but not to the same phenotypes that we examined in our study.

Some previous metabolomics studies used data reduction methods such as Principal Component Analysis (PCA) and Partial Least Squares Discriminant Analysis (PLS-DA) to summarize results. These are generally smaller studies with limited power to reliably estimate associations with individual metabolites. These methods can be used when reporting results from single studies, but are not suitable for meta-analysis results. Because these methods are dataset driven, results are not transportable from one dataset to another. For example, the first principle component (PC1) from a given study is not necessarily similar to PC1 from another. These methods must be run within a single dataset and do not accommodate meta-analyzed results and thus could not be used here.

Our study has limitations and strengths. The many metabolites analyzed represent only a fraction of the whole metabolome. Further, as is commonly done in these analyses, we only included metabolites with values reported for at least 25% of each population. Measurements were not done in the three datasets at the same time points. The number of metabolites reported by the laboratory increased over time. Thus, there are some metabolites that are not available (denoted as NA in tables) in one dataset or another. Further, it is possible that a given metabolite might meet threshold for inclusion in one dataset (nonmissing in <25%) but in another dataset perhaps 26% are missing, so it is coded as NA in the table. Our meta-analyses further excluded those with measurements only in one population. Thus, we may have missed many additional metabolites related to our phenotypes. We are limited in our ability to comprehensively assess how associations compared by ethnicity because analyses were mostly done in different analytic batches. The cross-sectional measures of lung function we analyzed reflect both the maximal growth by early adulthood and the rate of decline with age; we cannot disentangle these two processes. The three population-based samples had, as expected, small proportions of COPD cases and thus we have much more limited power to find associations with this disease phenotype compared to the quantitative spirometry traits. However, the number of individuals with COPD is larger than in most previous studies. We acknowledge that because our studies, like most general population cohorts, did not have post-bronchodilator measures of pulmonary function, our COPD definition does not meet GOLD criteria. However, bronchodilator testing for COPD diagnosis, has been shown to be poorly reproducible, especially in milder disease [53], the majority of COPD in our population. We also note that major recent studies of trajectories of lung function in COPD development used pre-bronchodilator measures [54] and large scale genetic studies of COPD have also defined disease based on pre-bronchodilator measures [55]. COPD in this general population study was less severe than in previous clinically-selected metabolomic studies. This enhances our ability to identify potential biomarkers of early disease processes, as opposed to effects of severe disease, including reduced protein and caloric intake, increased muscle turnover, and cachexia. Our relatively large population-based cohort samples enabled us to examine associations with metabolomic profiles across the range seen in the general population, unselected for any diseases. Further, classifying disease status within larger population-based studies enables comparison to the optimal representative control population that gave rise to the cases. We also studied populations from two different ancestral populations: European and African ancestries. Few data are available in non-European populations, and are able to find some common metabolic signatures for lung function.

In summary, in these two population-based cohorts, that span two continents and two ancestral populations, we both confirmed a number of previously reported associations with quantitative lung function traits and identified many novel ones. Many of these are also related to COPD. Patterns of the identified metabolites overlap with some previously reported for metabolic disorders, traits that are also associated with reduced lung function. These findings may shed light on mechanisms of reduction in lung function and its links to metabolic and cardiovascular disease. In addition, identifying subtle variation in circulating metabolites related to pulmonary function in the general population may identify circulating biomarkers of the early stages of COPD pathogenesis before spirometry has reduced to levels diagnostic of disease. Such biomarkers can help discover potential therapeutic interventions.

## 4. Material and Methods

### 4.1. Study Populations

The Atherosclerosis Risk in Communities (ARIC) study is an epidemiological study designed to investigate the etiology of cardiovascular disease in European and African Americans. ARIC enrolled 15,792 individuals aged 45–64 years from four U.S. communities (Forsyth County, NC; Jackson, MS; suburbs of Minneapolis, MN; and Washington County, MD) at baseline (1987–1989) [56]. Metabolite levels were measured in fasting serum samples collected at the baseline examination among a random sample of African and European Americans. Pulmonary function was measured at this same visit.

The KORA F4 study is a population-based cohort from Augsburg city and the two adjacent counties in southern Germany (KORA—Cooperative Health Research in the Region of Augsburg). Between 2006 and 2008, 3080 individuals aged 32 to 81 years participated in KORA F4, the seven-year follow-up of the KORA S4 survey. Details of the KORA platform and the study design have been described previously [57,58]. Pulmonary function was measured on a random sample of 1321 subjects aged 41 to 62 years and metabolite levels were measured in fasting blood samples collected at this visit.

### 4.2. Metabolome Measurements

In both studies, metabolites were assayed in serum by Metabolon Inc. (Durham, NC, USA) using a gas chromatography-mass spectrometry and liquid chromatography-mass spectrometry (GC-MS and LC-MS)-based metabolomic quantification protocol [59,60,61] in three different analytic waves, one wave per cohort (KORA (2009–2010), ARIC African-Americans (2010) and ARIC European-Americans (2014)). See Methods in Supplementary Materials for additional details.

### 4.3. Assessment of Pulmonary Function and Covariates

In ARIC, spirometry was conducted with a Collins Survey II water-seal spirometer (Collins Medical, Inc., Braintree, MA, USA) [62]. In KORA F4, spirometry was measured using a pneumotachograph-type spirometer (Masterscope PC, CareFusion/VIASYS Healthcare/Erich Jaeger, Höchberg, Germany). Height and weight were measured at the time of spirometry. Information on smoking was assessed by questionnaires. For both ARIC and KORA, predicted values were calculated using reference equations from the Global Lung Function Initiative [63]. COPD was defined using prebronchodilator spirometry applied to the Global Initiative for Chronic Obstructive Lung Disease (GOLD) criteria for diagnosis of COPD of at least moderate severity (FEV1 < 80% predicted and FEV1/FVC < 0.7) [55]. Estimated glomerular filtration rate (eGFR_CKD-EPI_) was calculated based on measured creatinine levels [64].

### 4.4. Human Subjects

ARIC was approved by the Institutional Review Board at the University of Minnesota, Johns Hopkins University, Wake Forest University, University of North Carolina, University of Texas Health Sciences Center at Houston, and University of Mississippi Medical Center. KORA was approved by the Ethics Committee of the Bavarian Medical Association (Bayerische Landesärztekammer) and the Bavarian commissioner for data protection and privacy (Bayerischer Datenschutzbeauftragter). Written consent was obtained from all ARIC and KORA study participants.

### 4.5. Statistical Analyses

In ARIC and KORA, metabolites with values reported for at least 25% of the study participants were analyzed. Metabolite missing values were imputed with half of the lowest value of the corresponding metabolite in each study by wave, and were standardized with mean at 0 and standard deviation at one (Methods in Supplementary Materials). The total number of metabolites included in the analyses was 721 for ARIC European Americans (measured in 2014), 355 for ARIC African Americans (measured in 2010) and 266 for KORA (measured 2009–2010). The reason for the differing number of metabolites in the three datasets is improvement in both instrumentation and the size of the authentic standard library over time.

We analyzed data from each of the 4742 study participants (1529 ARIC EA, 2354 ARIC AA and 859 KORA) with metabolite measurements and complete data on lung function and covariates. Linear regression models were fitted for each standardized metabolite and lung function parameter separately in each cohort, adjusting for the factors traditionally included in pulmonary function prediction equations (age, age^2^, sex, height, height^2^) [65], weight, smoking status (never, former, current as two categorical variables), lifetime cigarette pack years, cigarettes currently smoked per day, and the eGFR_CKD-EPI_ [64]. For the 368 metabolites detected in at least two of the three populations, study-specific associations with pulmonary outcomes were meta-analyzed using fixed effect inverse variance weighting [66]. Consequently, metabolites only available in one of the cohorts were excluded from the analyses. For correction for multiple testing we applied false discovery rate (FDR) of 0.05 using the Benjamini–Hochberg procedure and the stricter Bonferroni corrected *p* < 1.4 × 10^−4^ based on 368 tests, which assumes tests are independent. All analyses were performed using R, version 3.4.1 [67].

For metabolites associated with pulmonary function parameters at FDR < 0.05, we performed metabolic pathway analysis using MetaboAnalyst 3.0 [68] to examine if a particular pathway was represented more than expected compared to metabolic pathways based on the Human Metabolome Database 3.0 [69]. Statistical significance of enriched pathways was assessed by FDR < 0.05 using the hypergeometric test.

## Figures and Tables

**Table 1:** Demographics for African and European ancestry from the Atherosclerosis Risk in Communities (ARIC) study and Cooperative Health Research in the Region of Augsburg (KORA).

	ARIC African Ancestry	ARIC European Ancestry	KORA
N	2354	1529	859
Female, n (%)	1510 (64.1)	826 (54.0)	457 (53.2)
Age, years	53.0 (5.7)	54.6 (5.8)	53.8 (4.4)
Weight, kg	83.5 (17.0)	77.3 (16.3)	79.5 (17.0)
Height, cm	167.9 (8.9)	168.3 (9.6)	169.2 (9.2)
Smoking status: Never, n (%)	1172 (49.8)	610 (39.9)	319 (37.1)
Smoking status: Former, n (%)	523 (22.2)	527 (34.5)	360 (41.9)
Smoking status: Current, n (%)	659 (28.0)	392 (25.6)	180 (21.0)
Pack years, in ever smokers	21.9 (20.5)	29.2 (22.0)	22.8 (22.5)
Cigarettes per day, in current smokers	14.4 (9.6)	22.5 (12.6)	12.5 (9.3)
FEV1, mL	2519 (651)	2891 (777)	3268 (790)
FVC, mL	3286 (819)	3931 (991)	4220 (976)
% predicted FEV1	95.1 (17.1)	92.9 (16.6)	103.4 (16.1)
% predicted FVC	99.0 (15.7)	99.5 (14.5)	105.3 (13.7)
FEV1/FVC, %	76.8 (7.9)	73.6 (7.7)	77.5 (6.2)
eGFR, mL/min/1.73 m²	104.0 (18.3)	91.3 (14.5)	86.8 (13.4)
COPD * cases, n (%)	179 (7.6)	179 (11.7)	35 (4.1)
COPD severity classes *: Moderate	152 (6.5)	150 (9.8)	33 (3.9)
COPD severity classes *: Severe	25 (1.1)	24 (1.6)	2 (0.2)
COPD severity classes *: Very Severe	2 (0.1)	5 (0.3)	0 (0)
Diabetes, n (%)	413 (17.5)	126 (8.2)	36 (4.2)
Hypertension, n (%)	1258 (53.4)	469 (30.7)	277 (32.3)

Mean and SD provided unless otherwise noted. FEV1 represents forced expiratory volume in 1 s; FVC, forced vital capacity; eGFR, estimated glomerular filtration rate; and COPD, chronic obstructive pulmonary disease. * Defined based on prebronchodilator spirometry by FEV1/FVC < 0.7 and % predicted FEV1 < 80. COPD severity subclass definitions: moderate: % predicted 50 ≤ FEV1 < 80; severe % predicted 30 ≤ FEV1 < 50; very severe: FEV1 < 30 % predicted.

**Table 2:** Top 20 metabolites associated with FEV1 at Bonferroni significance (*p* < 1.4 × 10^−4^) in meta-analysis *.

		Meta-Analysis				ARIC African Ancestry (n = 2354)			ARIC European Ancestry (n = 1529)			KORA (n = 859)		
Metabolite	Super Pathway	Beta ^+^	SE	P	Direction **	Beta	SE	P	Beta	SE	P	Beta	SE	P
glycine	Amino Acid	38.8	7.0	3.6 × 10^−8^	+++	39.1	9.4	3.7 × 10^−5^	39.0	13.1	3.0 × 10^−3^	37.8	18.0	3.6 × 10^−2^
3-(4-hydroxyphenyl)lactate	Amino Acid	−37.2	7.7	1.4 × 10^−6^	−−−	−32.9	10.0	9.9 × 10^−4^	−26.9	15.2	7.6 × 10^−2^	−73.5	20.3	3.1 × 10^−4^
3-phenylpropionate (hydrocinnamate)	Amino Acid	32.7	6.8	1.5 × 10^−6^	+++	20.9	9.5	2.8 × 10^−2^	42.0	11.9	4.2 × 10^−4^	51.2	16.9	2.5 × 10^−3^
2-methylbutyrylcarnitine (C5)	Amino Acid	−36.2	7.5	1.6 × 10^−6^	−−−	−41.2	9.8	2.8 × 10^−5^	−38.0	15.0	1.2 × 10^−2^	−14.7	18.9	4.4 × 10^−1^
asparagine	Amino Acid	31.1	6.8	5.2 × 10^−6^	++−	29.8	9.2	1.2 × 10^−3^	54.0	12.7	2.2 × 10^−5^	−5.4	16.9	7.5 × 10^−1^
alpha-hydroxyisovalerate	Amino Acid	−30.9	7.0	8.9 × 10^−6^	−−−	−24.4	9.0	6.8 × 10^−3^	−32.2	13.9	2.1 × 10^−2^	−53.7	17.7	2.5 × 10^−3^
glutamine	Amino Acid	29.0	7.0	3.6 × 10^−5^	+++	32.5	9.6	7.3 × 10^−4^	35.2	12.9	6.7 × 10^−3^	7.4	17.0	6.6 × 10^−1^
isoleucine	Amino Acid	−28.9	7.4	1.0 × 10^−4^	−−−	−28.9	9.4	2.2 × 10^−3^	−34.8	14.5	1.7 × 10^−2^	−15.3	21.8	4.8 × 10^−1^
serotonin (5HT)	Amino Acid	26.2	6.9	1.4 × 10^−4^	+++	33.2	9.0	2.4 × 10^−4^	14.0	13.4	3.0 × 10^−1^	20.5	17.2	2.3 × 10^−1^
glycerate	Carbohydrate	31.7	7.0	5.8 × 10^−6^	++−	39.2	9.3	2.8 × 10^−5^	43.8	13.5	1.2 × 10^−3^	−11.0	16.7	5.1 × 10^−1^
lactate	Carbohydrate	−30.4	6.9	1.1 × 10^−5^	−−−	−27.7	9.3	2.9 × 10^−3^	−36.4	12.7	4.1 × 10^−3^	−28.4	17.7	1.1 × 10^−1^
fructose	Carbohydrate	−27.6	6.7	3.8 × 10^−5^	−−−	−28.6	8.5	8.2 × 10^−4^	−39.2	14.3	6.3 × 10^−3^	−8.2	16.7	6.2 × 10^−1^
mannose	Carbohydrate	−27.4	7.0	9.9 × 10^−5^	−−−	−33.1	8.9	2.0 × 10^−4^	−21.2	14.9	1.5 × 10^−1^	−12.5	18.1	4.9 × 10^−1^
glycerol	Lipid	−36.4	7.2	4.7 × 10^−7^	−−−	−36.5	9.5	1.3 × 10^−4^	−43.0	14.0	2.1 × 10^−3^	−24.8	18.2	1.7 × 10^−1^
7-alpha-hydroxy-3-oxo-4-cholestenoate (7-Hoca)	Lipid	−30.4	6.9	9.5 × 10^−6^	−−−	−18.6	9.4	4.8 × 10^−2^	−33.0	12.5	8.2 × 10^−1^	−63.7	16.9	1.8 × 10^−4^
N2,N2-dimethylguanosine	Nucleotide	−32.3	7.2	6.4 × 10^−6^	−−−	−22.6	9.7	2.0 × 10^−2^	−58.5	13.6	1.9 × 10^−5^	−21.6	16.9	2.0 × 10^−1^
pseudouridine	Nucleotide	−33.8	7.6	8.9 × 10^−6^	−−−	−29.9	10.1	3.1 × 10^−3^	−21.8	14.9	1.4 × 10^−1^	−65.3	18.4	4.1 × 10^−4^
gamma-glutamylthreonine	Peptide	36.3	6.8	1.0 × 10^−7^	+++	34.2	8.8	1.1 × 10^−4^	52.6	13.9	1.6 × 10^−4^	20.0	16.8	2.4 × 10^−1^
gamma-glutamylleucine	Peptide	34.8	7.0	5.6 × 10^−7^	+++	34.4	8.8	9.6 × 10^−5^	40.6	13.6	2.9 × 10^−3^	23.7	20.5	2.5 × 10^−1^
gamma-glutamylvaline	Peptide	31.8	6.9	4.1 × 10^−6^	+++	30.9	8.8	4.8 × 10^−4^	43.2	13.4	1.3 × 10^−3^	11.6	19.9	5.6 × 10^−1^

* Top 20 metabolites, based on meta-analysis *p* value, measured in all three populations are shown in this table; a total of 30 met Bonferroni correction. All 95 metabolites associated with FEV1 at FDR < 0.05 are shown in Supplementary Table S1. + Regression coefficient: mL difference in FEV1 per SD change of the metabolite levels. ** Direction of effect: the first, second and third +, or − refers to the direction of effect in ARIC African ancestry, ARIC European ancestry and KORA.

**Table 3:** Top 20 metabolites associated with FVC at Bonferroni significance (*p* < 1.4 × 10^−4^) in meta-analysis *.

		Meta-Analysis				ARIC African-Ancestry (n = 2354)			ARIC European Ancestry (n = 1529)			KORA (n = 859)		
Metabolite	Super Pathway	Beta ^+^	SE	P	Direction **	Beta	SE	P	Beta	SE	P	Beta	SE	P
glycine	Amino Acid	56.9	8.0	9.6 × 10^−13^	+++	58.4	10.9	8.9 × 10^−8^	61.2	14.7	3.4 × 10^−5^	44.6	19.3	2.1 × 10^−2^
isoleucine	Amino Acid	−53.8	8.4	1.7 × 10^−10^	−−−	−47.0	10.9	1.6 × 10^−5^	−69.7	16.3	2.0 × 10^−5^	−52.0	23.2	2.5 × 10^−2^
N-acetylglycine	Amino Acid	44.3	7.9	2.4 × 10^−8^	+++	43.8	11.1	8.5 × 10^−5^	49.8	14.3	5.0 × 10^−4^	36.4	18.8	5.3 × 10^−2^
asparagine	Amino Acid	41.6	7.7	7.0 × 10^−8^	+++	42.7	10.6	6.0 × 10^−5^	62.6	14.3	1.3 × 10^−5^	4.4	18.1	8.1 × 10^−1^
2-methylbutyrylcarnitine (C5)	Amino Acid	−44.9	8.5	1.5 × 10^−7^	−−−	−44.5	11.3	9.0 × 10^−5^	−55.9	16.9	9.7 × 10^−4^	−30.2	20.2	1.4 × 10^−1^
glutamine	Amino Acid	39.0	7.9	9.2 × 10^−7^	+++	37.2	11.1	8.0 × 10^−4^	56.3	14.6	1.1 × 10^−4^	16.5	18.2	3.7 × 10^−1^
3-(4-hydroxyphenyl)lactate	Amino Acid	−42.6	8.7	1.1 × 10^−6^	−−−	−35.2	11.5	2.3 × 10^−3^	−32.9	17.1	5.4 × 10^−2^	−84.3	21.7	1.1 × 10^−4^
3-phenylpropionate (hydrocinnamate)	Amino Acid	36.3	7.7	2.3 × 10^−6^	+++	24.1	11.0	2.8 × 10^−2^	42.0	13.4	1.8 × 10^−3^	59.2	18.1	1.1 × 10^−3^
tyrosine	Amino Acid	−35.2	8.0	1.2 × 10^−5^	−−−	−24.5	10.5	2.0 × 10^−2^	−42.8	16.2	8.2 × 10^−3^	−61.6	19.6	1.8 × 10^−3^
valine	Amino Acid	−33.9	8.1	3.1 × 10^−5^	−−−	−36.4	10.5	5.3 × 10^−4^	−33.7	16.3	3.9 × 10^−2^	−24.2	21.2	2.5 × 10^−1^
phenylalanine	Amino Acid	−33.2	8.0	3.3 × 10^−5^	−−−	−26.2	10.4	1.2 × 10^−2^	−37.3	16.2	2.2 × 10^−2^	−52.3	19.7	8.2 × 10^−3^
5-oxoproline	Amino Acid	31.4	7.8	5.9 × 10^−5^	++−	42.8	10.6	5.7 × 10^−5^	44.7	14.9	2.7 × 10^−3^	−22.4	18.2	2.2 × 10^−1^
mannose	Carbohydrate	−41.1	8.0	2.5 × 10^−7^	−−−	−52.1	10.2	3.8 × 10^−7^	−39.4	16.8	1.9 × 10^−2^	−3.6	19.4	8.5 × 10^−1^
lactate	Carbohydrate	−38.4	7.8	8.9 × 10^−7^	−−−	−33.6	10.7	1.8 × 10^−3^	−45.8	14.3	1.4 × 10^−3^	−40.3	18.9	3.3 × 10^−2^
fructose	Carbohydrate	−32.3	7.6	2.2 × 10^−5^	−−−	−39.5	9.8	6.2 × 10^−5^	−31.9	16.2	4.9 × 10^−2^	−9.0	17.9	6.2 × 10^−1^
glucose	Carbohydrate	−30.7	7.7	6.3 × 10^−5^	−−−	−37.1	9.5	9.7 × 10^−5^	−21.0	18.0	2.4 × 10^−1^	−16.1	18.7	3.9 × 10^−1^
biliverdin	Cofactors and Vitamins	31.0	7.9	8.6 × 10^−5^	+++	31.0	11.5	7.1 × 10^−3^	28.0	13.5	3.8 × 10^−2^	36.2	18.1	4.6 × 10^−2^
glycerol	Lipid	−33.4	8.2	4.6 × 10^−5^	−−−	−39.1	11.0	4.0 × 10^−4^	−36.1	15.7	2.2 × 10^−2^	−11.4	19.5	5.6 × 10^−1^
pseudouridine	Nucleotide	−38.5	8.6	7.8 × 10^−6^	−−−	−35.4	11.7	2.4 × 10^−3^	−29.6	16.7	7.7 × 10^−2^	−59.8	19.8	2.6 × 10^−3^
urate	Nucleotide	−35.3	8.3	2.1× 10^−5^	−−−	−38.2	11.1	5.9 × 10^−4^	−26.2	14.8	7.7 × 10^−2^	−45.3	23.5	5.4 × 10^−2^

* Top 20 metabolites (based on meta-analysis *p* value) measured in all three populations are shown in this table: a total of 37 met Bonferroni correction. All 100 metabolites associated with FVC at FDR < 0.05 were shown in Supplementary Table S2. ^+^ Regression coefficient: mL difference in FVC per SD change of the metabolite levels. ** Direction of effect: the first, second and third + or − refers to the direction of effect in ARIC African ancestry, ARIC European ancestry and KORA.

**Table 4:** Metabolites associated with FEV1/FVC at FDR < 0.05 in meta-analysis *.

		Meta-Analysis				ARIC African Ancestry (n = 2354)			ARIC European Ancestry (n = 1529)			KORA (n = 859)		
Metabolite	Super Pathway	Beta ^+^	SE	P	Direction **	Beta	SE	P	Beta	SE	P	Beta	SE	P
3-methoxytyrosine	Amino Acid	−0.42	0.10	5.0 × 10^−5^	−−−	−0.37	0.13	4.1 × 10^−3^	−0.45	0.35	2.0 × 10^−1^	−0.53	0.20	8.1 × 10^−3^
androsterone sulfate	Lipid	0.37	0.10	2.5 × 10^−4^	+++	0.29	0.16	7.9 × 10^−2^	0.31	0.17	7.0 × 10^−2^	0.57	0.20	5.6 × 10^−3^
glycerol	Lipid	−0.36	0.11	8.6 × 10^−4^	−−−	−0.27	0.16	9.1 × 10^−2^	−0.52	0.20	7.4 × 10^−3^	−0.32	0.22	1.4 × 10^−1^
dehydroisoandrosterone sulfate (DHEA-S)	Lipid	0.35	0.11	1.2 × 10^−3^	+++	0.39	0.17	2.1 × 10^−2^	0.37	0.19	5.1 × 10^−2^	0.27	0.22	2.2 × 10^−1^
lathosterol	Lipid	0.34	0.10	7.4 × 10^−4^	+++	0.38	0.15	1.3 × 10^−2^	0.37	0.18	4.1 × 10^−2^	0.24	0.20	2.5 × 10^−1^
oleoylcarnitine	Lipid	−0.42	0.10	2.9 × 10^−5^	−−−	−0.46	0.15	2.1 × 10^−3^	−0.50	0.18	4.8 × 10^−3^	−0.23	0.20	2.7 × 10^−1^
7-alpha-hydroxy-3-oxo-4-cholestenoate (7-Hoca)	Lipid	−0.33	0.10	9.6 × 10^−4^	−−−	−0.32	0.16	3.8 × 10^−2^	−0.28	0.17	1.1 × 10^−1^	−0.42	0.21	4.0 × 10^−2^
theophylline	Xenobiotics	−0.62	0.10	4.0 × 10^−10^	−−−	−0.72	0.14	4.8 × 10^−7^	−0.73	0.18	7.5 × 10^−5^	−0.26	0.21	2.1 × 10^−1^

* Metabolites measured in all three populations were shown in this table. All metabolites associations with FEV1/FVC were shown in Supplementary Table S6. ^+^ Regression coefficient: difference in FEV1/FVC (scaled as a percentage) per SD change in the metabolite levels. ** Direction of effect: the first, second and third +, or − refers to the direction of effect in ARIC African ancestry, ARIC European ancestry and KORA.

**Table 5:** Pathway enriched for FEV1 and FVC related metabolites.

Trait	Pathway	Analytes in the Pathway, N	Analytes with FDR < 0.05, N	Analytes with FDR < 0.05	P	FDR
FEV1	Aminoacyl-tRNA biosynthesis	75	8	asparagine, phenylalanine, glutamine, cysteine, glycine, isoleucine, threonine, tyrosine	3.3 × 10^−4^	0.02
FEV1	Phenylalanine metabolism	45	6	phenylalanine, hydrocinnamic acid, hippuric acid, succinic acid, n-acetyl-phenylalanine, tyrosine	5.9 × 10^−4^	0.02
FEV1	Nitrogen metabolism	39	5	phenylalanine, tyrosine, asparagine, glutamine, glycine	2.1× 10^−3^	0.05
FEV1	Alanine, aspartate and glutamate metabolism	24	4	asparagine, oxoglutaric acid, glutamine, succinic acid	2.3 × 10^−3^	0.05
FVC	Aminoacyl-tRNA biosynthesis	75	9	asparagine, phenylalanine, glutamine, glycine, valine, isoleucine, leucine, threonine, tyrosine.	7.0 × 10^−5^	0.006
FVC	Phenylalanine metabolism	45	6	phenylalanine, hydrocinnamic acid, hippuric acid, succinic acid, n-acetyl-phenylalanine, tyrosine	7.1 × 10^−4^	0.03

**Table 6:** Metabolites associated with COPD at FDR < 0.05 in meta-analysis *.

		Meta-analysis			ARIC African Ancestry (n = 2354)		ARIC European Ancestry (n = 1529)		KORA (n = 859)	
Metabolite	Super Pathway	OR ^+^ (95% CI)	P	Direction **	OR (95% CI)	P	OR (95% CI)	P	OR (95% CI)	P
3-(4-hydroxyphenyl)lactate	Amino Acid	1.28 (1.14, 1.44)	3.6 × 10^−5^	+++	1.28 (1.09, 1.51)	3.2 × 10^−3^	1.13 (0.93, 1.38)	2.3 × 10^−1^	1.76 (1.28, 2.42)	4.6 × 10^−4^
3-methoxytyrosine	Amino Acid	1.19 (1.1, 1.3)	4.1 × 10^−5^	+++	1.19 (1.08, 1.3)	2.5 × 10^−4^	1.11 (0.79, 1.55)	5.4 × 10^−1^	1.36 (1, 1.85)	4.6 × 10^−2^
homocitrulline	Amino Acid	1.21 (1.08, 1.36)	8.5 × 10^−4^	++−	1.26 (1.09, 1.47)	2.5 × 10^−3^	1.21 (1, 1.47)	5.3 × 10^−2^	0.95 (0.66, 1.39)	8.1 × 10^−1^
serotonin (5HT)	Amino Acid	0.83 (0.74, 0.93)	1.7 × 10^−3^	−−−	0.8 (0.67, 0.94)	7.7 × 10^−3^	0.87 (0.72, 1.05)	1.4 × 10^−1^	0.82 (0.57, 1.19)	3.0 × 10^−1^
ornithine	Amino Acid	1.2 (1.07, 1.34)	1.9 × 10^−3^	+++	1.06 (0.9, 1.24)	5.0 × 10^−1^	1.4 (1.17, 1.68)	2.7 × 10^−4^	1.21 (0.85, 1.73)	2.8 × 10^−1^
glycerate	Carbohydrate	0.8 (0.71, 0.91)	3.3 × 10^−4^	−−+	0.77 (0.65, 0.92)	3.9 × 10^−3^	0.77 (0.64, 0.92)	5.2 × 10^−3^	1.12 (0.78, 1.61)	5.4 × 10^−1^
succinylcarnitine	Energy	1.21 (1.08, 1.36)	1.4 × 10^−3^	+++	1.17 (1, 1.37)	5.3 × 10^−2^	1.32 (1.08, 1.6)	6.3 × 10^−3^	1.09 (0.74, 1.61)	6.7 × 10^−1^
oleoylcarnitine	Lipid	1.22 (1.1, 1.35)	1.6 × 10^−4^	+++	1.28 (1.11, 1.48)	6.1 × 10^−4^	1.14 (0.96, 1.35)	1.3 × 10^−1^	1.2 (0.89, 1.62)	2.3 × 10^−1^
5-dodecenoate (12:1n7)	Lipid	1.23 (1.1, 1.37)	1.9 × 10^−4^	+++	1.27 (1.09, 1.48)	1.7 × 10^−3^	1.14 (0.96, 1.36)	1.5 × 10^−1^	1.33 (0.97, 1.81)	7.7 × 10^−2^
docosahexaenoate (DHA, 22:6n3)	Lipid	0.79 (0.7, 0.9)	2.6 × 10^−4^	−−+	0.85 (0.72, 1)	4.6 × 10^−2^	0.63 (0.51, 0.78)	3.3 × 10^−5^	1.11 (0.78, 1.6)	5.5 × 10^−1^
androsterone sulfate	Lipid	0.81 (0.71, 0.91)	8.2 × 10^−4^	−−−	0.9 (0.75, 1.08)	2.4 × 10^−1^	0.79 (0.66, 0.95)	1.4 × 10^−2^	0.36 (0.21, 0.63)	2.8 × 10^−4^
7-alpha-hydroxy-3-oxo-4-cholestenoate (7-Hoca)	Lipid	1.19 (1.07, 1.33)	1.0 × 10^−3^	+++	1.15 (0.98, 1.35)	9.3 × 10^−2^	1.22 (1.04, 1.43)	1.3 × 10^−2^	1.29 (0.94, 1.76)	1.2 × 10^−1^
glycerol	Lipid	1.21 (1.08, 1.36)	1.0 × 10^−3^	+++	1.21 (1.02, 1.42)	2.4 × 10^−2^	1.2 (0.99, 1.45)	6.4 × 10^−2^	1.28 (0.94, 1.75)	1.2 × 10^−1^
pseudouridine	Nucleotide	1.23 (1.08, 1.39)	1.2 × 10^−3^	+++	1.23 (1.04, 1.44)	1.5 × 10^−2^	1.22 (0.98, 1.53)	7.7 × 10^−2^	1.27 (0.86, 1.86)	2.3 × 10^−1^
theophylline	Xenobiotics	1.26 (1.17, 1.36)	1.3 × 10^−9^	+++	1.25 (1.14, 1.37)	3.1 × 10^−6^	1.25 (1.09, 1.45)	2.0 × 10^−3^	1.39 (1.07, 1.81)	1.4 × 10^−2^
1-methylurate	Xenobiotics	1.21 (1.1, 1.33)	1.3 × 10^−4^	+++	1.22 (1.07, 1.4)	3.7 × 10^−3^	1.23 (1.06, 1.44)	7.6 × 10^−3^	1.04 (0.75, 1.43)	8.3 × 10^−1^

* Metabolites measured in all three populations were shown in this table. All metabolites associations with COPD were shown in Supplementary Table S7. ^+^ Odds ratio: difference in COPD odds per SD change in the metabolite levels. ** Direction of effect: the first, second and third +, or − refers to the direction of effect in ARIC African ancestry, ARIC European ancestry and KORA.

## Data Availability

Primary data is available through the established application procedures for the ARIC and KORA studies. Application for KORA data can be made via the KORA Project Application Self-Service Tool, KORA.PASST, at https://epi.helmholtz-muenchen.de/.

## Supplementary Materials

The following are available online at https://www.mdpi.com/2218-1989/9/4/61/s1, Table S1. Metabolites associated with FEV1 at FDR < 0.05 in fixed effect meta-analysis; Table S2. Metabolites associated with FVC at FDR < 0.05 in fixed effect meta-analysis; Table S3. Metabolites associated with FEV1 and/or FVC at FDR < 0.05 and their association with COPD; Table S4. Associations between metabolites and FEV1; Table S5. Associations between metabolites and FVC; Table S6. Associations between metabolites and FEV1/FVC; Table S7. Associations between metabolites and COPD; Table S8. Metabolites where the direction of effect differed between the meta-analysis result and the result in a component study.
