# Supplementary material for: Metabolomics Identifies Novel Blood Biomarkers of Pulmonary Function and COPD in the General Population
Source: Metabolites. 2019 Apr 1;9(4):61. doi: 10.3390/metabo9040061 (PMC6523962; doi:10.3390/metabo9040061)
Supplement: Supplementary file 1 [file metabolites-09-00061-s001.zip › Supplemental_Methods.pdf]

SUPPLEMENTAL MATERIAL

Supplemental Methods

Sample Selection

In ARIC, a random set of 1880 samples from 4266 African-American was selected for metabolome measure at 2009 and an additional set of 599 African-Americans and 1553 European-Americans was select for metabolome measure at 2014 with the priority on participants with existing genotype information. In KORA, cross-sectional health surveys were performed in about 430,000 inhabitants aged 25 to 74 years with German nationality in the study region of Augsburg. Samples were drawn in a two-stage procedure where Augsburg city and sixteen communities from adjacent counties were selected by cluster sampling and then stratified random sampling was performed within each community. By this means, four cross-sectional health surveys S1 to S4, each comprising of an independent random sample, have been performed at five years intervals.

Blood Collection

The ARIC protocol for blood sample collection and handling were designed to minimize spontaneous biochemical reactions after blood collection following standard ARIC lab protocols. In brief, most blood was drawn after a period of at least 12 hours overnight fasting to avoid variation. Blood samples were immediately put in an ice water bath after venipuncture. Centrifugation was then performed within ten minutes after venipuncture at room temperature (15-25°C). After centrifugation, the aliquots were stored at -80°C within 90 minutes from venipuncture. For KORA, blood was drawn after a period of at least 10 hours overnight fasting to avoid variation. Material was drawn into serum gel tubes, inverted two times and then allowed to rest for 30 minutes at room temperature (18-25°C) to obtain complete coagulation. The material was then centrifuged for 10 minutes (2,750g at 15°C). Serum was divided into aliquots and kept for a maximum of 6 hours at 4°C, after which it was deep frozen to -80°C until analysis. Serum samples that were never thawed were used in both ARIC and KORA for metabolomics analysis to minimize the effects of freeze-thaw on metabolite levels.

Sample Preparation for Global Metabolomics

Non-targeted mass spectroscopy (MS) analysis was performed at Metabolon, Inc. Samples were stored at –80°C until processed. Sample preparation was carried out as described previously at Metabolon, Inc.[1]. Briefly, recovery standards were added prior to the first step in the extraction process for quality control purposes. To remove protein, dissociate small molecules bound to protein or trapped in the precipitated protein matrix, and to recover chemically diverse metabolites, proteins were precipitated with methanol under vigorous shaking for 2 min (Glen Mills Genogrinder 2000) followed by centrifugation. The resulting extract was divided into fractions and vacuum dried. For each sample, dried extracts were redissolved in injection solvent containing eight or more injection standards at fixed concentrations, depending on the platform, to assure injection and chromatographic consistency.

Three types of controls were analyzed in concert with the experimental samples: samples generated from a pool of human plasma extensively characterized by Metabolon, Inc. served as technical replicate throughout the data set; extracted water samples served as process blanks; and a cocktail of standards spiked into every analyzed sample allowed instrument performance monitoring. Instrument variability was determined by calculating the median relative standard deviation (RSD) for the standards that were added to each sample prior to injection into the mass spectrometers (median RSD = 2-5%; $n \geq 30$ standards). Overall process variability was determined by calculating the median RSD for all endogenous metabolites (i.e., non-instrument standards) present in 100% of the pooled human plasma samples (median RSD = 7-10%; n = several hundred metabolites). Experimental samples and controls were randomized across the platform run.

Mass Spectrometry Analysis

Each sample was analyzed using non-targeted gas chromatography-mass spectrometry (GC-MS) [2] and ultra-performance liquid chromatography-tandem mass spectrometry (UPLC-MS/MS) [1]. Samples for the KORA study were analyzed in a single batch at Metabolon, Inc. in 2009, while the ARIC study samples were processed and analyzed in two batches, one in 2010 and the other in 2014. Extract fractions destined for analysis by GC-MS were derivatized under nitrogen using bistrimethyl-silyltrifluoroacetamide. Derivatized samples were separated on a 5% phenyldimethyl silicone column with helium as carrier gas and a temperature ramp from 60° to 340°C within a 17-min period. All samples were analyzed on a Thermo-Finnigan Trace DSQ MS operated at unit mass resolving power with electron impact (EI) ionization and a 50–750 atomic mass unit scan range.

Extracts from the KORA and 2010 ARIC study samples were analyzed by a UPLC-MS/MS platform that consisted of a Waters Acquity UPLC and a ThermoFisher LTQ mass spectrometer, which included an electrospray ionization source and a linear ion-trap mass analyzer operated at nominal mass resolution. The instrumentation was set to monitor for positive ions in acidic extracts or negative ions in basic extracts through independent injections. Reconstituted extracts were loaded onto columns (Waters UPLC BEH C18-2.1×100 mm, 1.7 µm), and gradient-eluted with water and 95% methanol containing 0.1% formic acid (acidic extracts) or 6.5 mM ammonium bicarbonate (basic extracts). Columns were washed and reconditioned after every injection. The instrument was set to scan 99–1000 m/z and alternated between MS and data-dependent MS2 scans using dynamic exclusion. The scan speed was approximately six scans per s (three MS and three MS/MS scans).

Extracts from the 2014 ARIC study samples were analyzed by a UPLC-MS/MS platform that consisted of a Waters ACQUITY UPLC and a Thermo Scientific Q-Exactive high resolution/accurate mass spectrometer interfaced with a heated electrospray ionization (HESI-II) source and Orbitrap mass analyzer operated at 35,000 mass resolution. One aliquot was analyzed using acidic positive ion-optimized conditions in which the extract was gradient eluted from a C18 column (Waters UPLC BEH C18-2.1x100 mm, 1.7 µm) using water and methanol containing 0.1% formic acid. A second aliquot was analyzed using basic negative ion-optimized conditions using a separate dedicated C18 column. The basic extracts were gradient eluted from the column using methanol and water, however with 6.5mM Ammonium Bicarbonate at pH 8. A third aliquot was analyzed via negative ionization following elution from a Hydrophilic Interaction Liquid Chromatography (HILIC) column (Waters UPLC BEH Amide 2.1x150 mm, 1.7 µm) using a gradient consisting of water and acetonitrile with 10mM Ammonium Formate, pH 10.8. Columns were washed and reconditioned after every injection. The MS analysis alternated between MS and data-dependent MSn scans using dynamic exclusion. The scan range varied slighted between methods but covered 70-1000 m/z.

Compound Identification, Quantification, and Data Curation

Metabolites were identified by automated comparison of the ion features in the experimental samples to a reference library of chemical standard entries that included retention time, molecular weight (m/z), preferred adducts, and in-source fragments as well as associated MS spectra and curated by visual inspection for quality control using software developed at Metabolon [3]. Identification of known chemical entities is based on comparison to Metabolon's spectral library of >4,000 purified chemical standards. Commercially available purified standard compounds were acquired and registered into LIMS for distribution to the various GC-MS and UPLC-MS/MS platforms for determination of their detectable characteristics. Known metabolites reported in this study conform to confidence Level 1 (the highest confidence level of identification) of the Metabolomics Standards Initiative [4,5], unless otherwise denoted with an asterisk. An additional 5,300 mass spectral entries have been created for structurally unnamed biochemicals, which have been identified by virtue of their recurrent nature (both chromatographic and mass spectral). These compounds have the potential to be identified by future acquisition of a matching purified standard or by classical structural analysis.

Peaks were quantified using area-under-the-curve. Raw area counts for each metabolite in each sample were normalized to correct for variation resulting from instrument inter-day tuning differences by the median value for each run-day, therefore, setting the medians to 1.0 for each run [6]. This preserved variation between samples but allowed metabolites of widely different raw peak areas to be compared on a similar graphical scale.

Metabolomics Measurements

In ARIC, the batch in 2010 included 1880 African Americans and resulted in a total of 384 named metabolites detected and semi-quantified; 355 of 384 metabolites had detectable measurement for at least 25% of individuals. The batch in 2014 included 599 African Americans and 1553 European Americans with a total of 787 named metabolites detected and semi-quantified; and 721 out of 787 metabolites had metabolites had detectable measurement for at least 25%. In KORA, a total of 292 known metabolites were measured in serum samples from 1768 participants. Of 292 metabolites, 266 had detectable measurement for at least 25% of individuals.

Cotinine Imputation

Cotinine is a well-established biomarker of recent smoking, therefore, half of the lowest value was assigned to people who self-reported being current smokers but had missing cotinine values, and zero was assigned to people with missing values who self-reported being non-smokers or past smokers.

References:

1. Evans, A.M.; DeHaven, C.D.; Barrett, T.; Mitchell, M.; Milgram, E. Integrated, nontargeted ultrahigh performance liquid chromatography/electrospray ionization tandem mass spectrometry platform for the identification and relative quantification of the small-molecule complement of biological systems. *Anal Chem* **2009**, *81*, 6656-6667, doi:10.1021/ac901536h.
2. Sha, W.; da Costa, K.A.; Fischer, L.M.; Milburn, M.V.; Lawton, K.A.; Berger, A.; Jia, W.; Zeisel, S.H. Metabolomic profiling can predict which humans will develop liver dysfunction when deprived of dietary choline. *FASEB J* **2010**, *24*, 2962-2975, doi:10.1096/fj.09-154054.
3. Dehaven, C.D.; Evans, A.M.; Dai, H.; Lawton, K.A. Organization of GC/MS and LC/MS metabolomics data into chemical libraries. *J Cheminform* **2010**, *2*, 9, doi:10.1186/1758-2946-2-9.
4. Schrimpe-Rutledge, A.C.; Codreanu, S.G.; Sherrod, S.D.; McLean, J.A. Untargeted Metabolomics Strategies-Challenges and Emerging Directions. *J Am Soc Mass Spectrom* **2016**, *27*, 1897-1905, doi:10.1007/s13361-016-1469-y.
5. Sumner, L.W.; Amberg, A.; Barrett, D.; Beale, M.H.; Beger, R.; Daykin, C.A.; Fan, T.W.; Fiehn, O.; Goodacre, R.; Griffin, J.L., et al. Proposed minimum reporting standards for chemical analysis Chemical Analysis Working Group (CAWG) Metabolomics Standards Initiative (MSI). *Metabolomics* **2007**, *3*, 211-221, doi:10.1007/s11306-007-0082-2.
6. Wulff, J.E.; Mitchell, M.W. A Comparison of Various Normalization Methods for LC/MS Metabolomics Data. *Adv Biosci Biotech* **2018**, 9,339-351, doi: 10.4236/abb.2018.98022.
